# Supplementary material for: Nanopore long-read RNAseq reveals widespread transcriptional variation among the surface receptors of individual B cells
Source: Nat Commun. 2017 Jul 19;8:16027. doi: 10.1038/ncomms16027 (PMC5524981; doi:10.1038/ncomms16027)
Supplement: Supplementary Information [file ncomms16027-s1.pdf]

File name: Supplementary Information
Description: Supplementary figures and supplementary tables.

File name: Peer review file
Description:

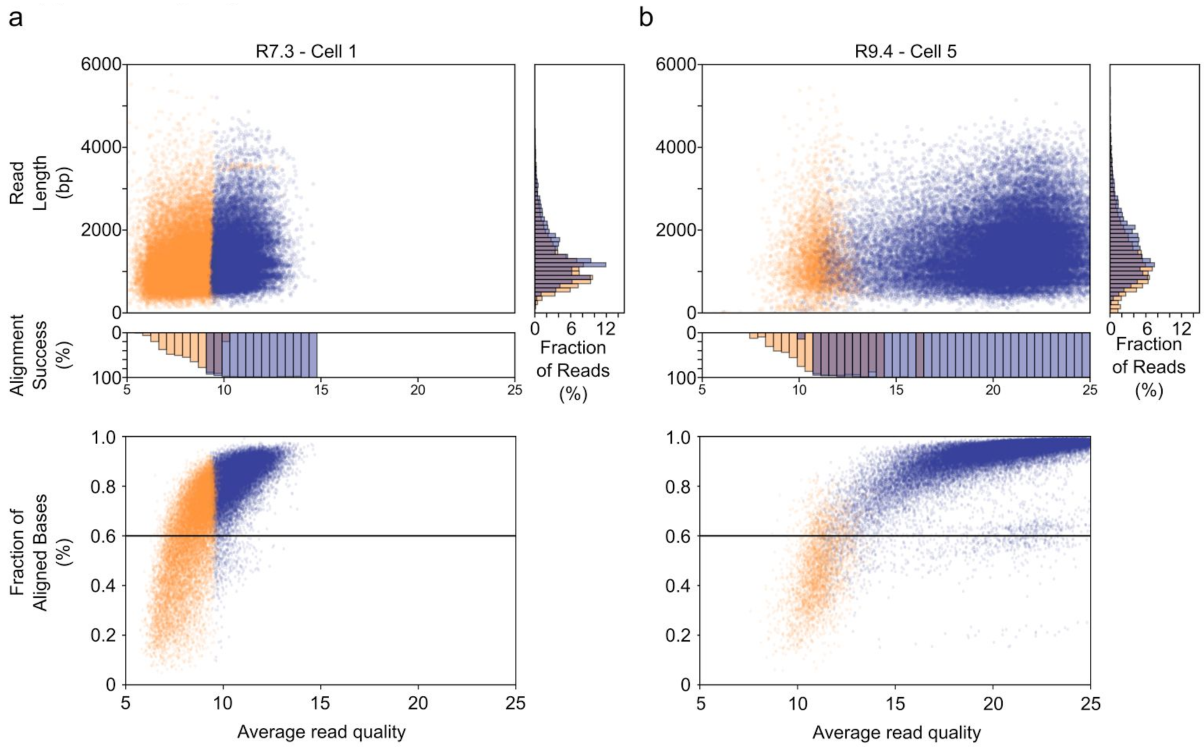

Supplementary Fig. 1: Sequencing run characteristics

The scatter plots on top shows read length and average sequence quality for ONT 2D reads that passed (blue) or failed (orange) the Metrichor analysis pipeline quality threshold in individual R7.3 (left) and R9.4 (right) sequencing runs. The histograms on the right of the scatter plots show the reads binned by read length using the same colors as the plot in the center to indicate passed (blue) or failed (orange). Using the same color scheme, the histogram at the bottom shows alignment success (percent of reads successfully aligned by BLAT) for reads binned by sequence quality score.

The scatter plot on the bottom shows ONT 2D reads successfully aligned by BLAT. The ratio of aligned/total bases of each read (blue=pass, orange=fail) plotted against average sequence quality score. The alignment quality cut-off of 60% aligned bases is shown as a black line.

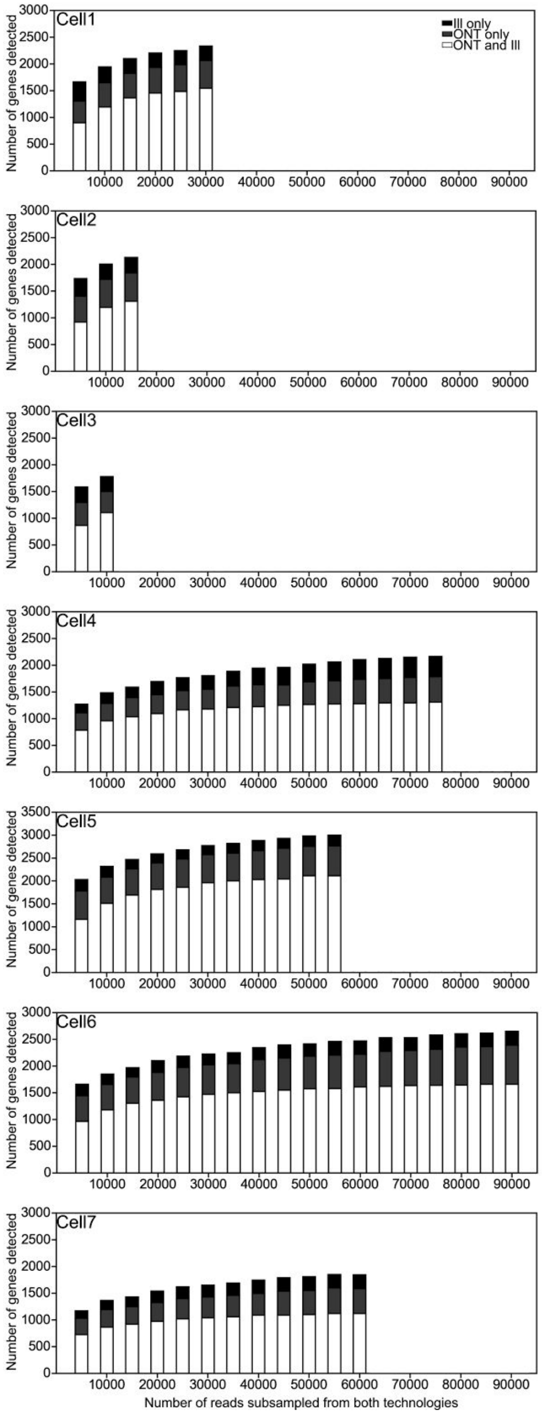

Supplementary Fig. 2: Gene detection with subsampled data

For each cell Illumina and ONT reads were subsampled in 5000 read bins until either the total number of Illumina or ONT reads was reached. The subsampled reads were used to quantify gene expression. Genes with a RPG10K value >0, i.e. with a single mapped read were scored as detected (x-axis = # of genes detected, y-axis= # of reads subsampled). Reads detected in both or either technology are shown in different colored bars.

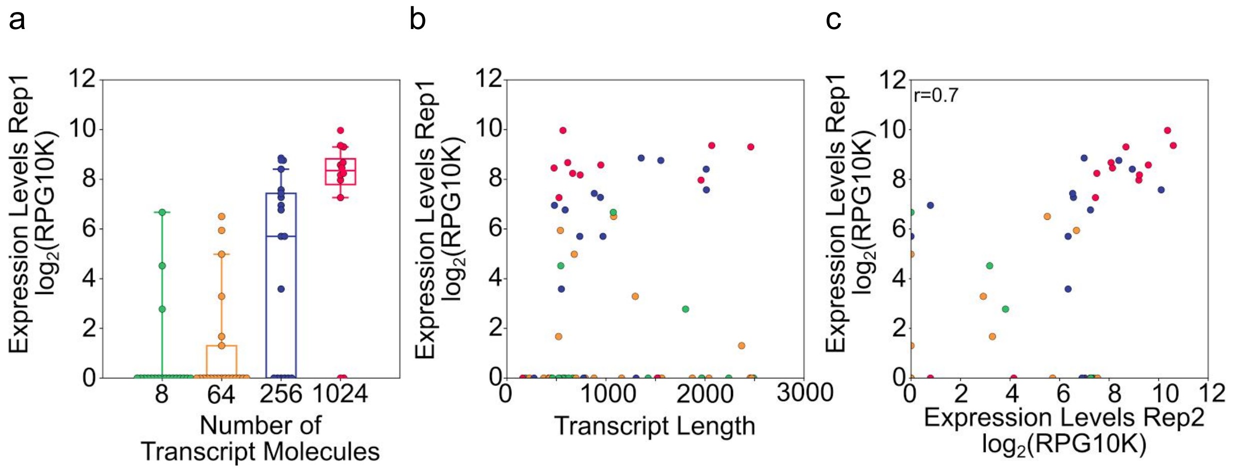

Supplementary Fig. 3: Quantifying SIRV transcripts amplified from 10fg starting material with ONT RNAseq

a) SIRV transcript levels of Replicate 1 (Rep1: 10fg SIRV pool E2) as measured with ONT RNAseq. Transcripts are binned by their starting molecule numbers. b) SIRV transcript levels of Replicate 1 are plotted against transcript length with colors corresponding to groups in a).

c) Scatter plot showing correlation of SIRV transcript expression levels of Replicate 1 (Rep1: 10fg SIRV pool E2) and Replicate 2 (Rep2: 10fg SIRV pool E2) , both measured by ONT RNAseq r-value is shown as Pearson-r.

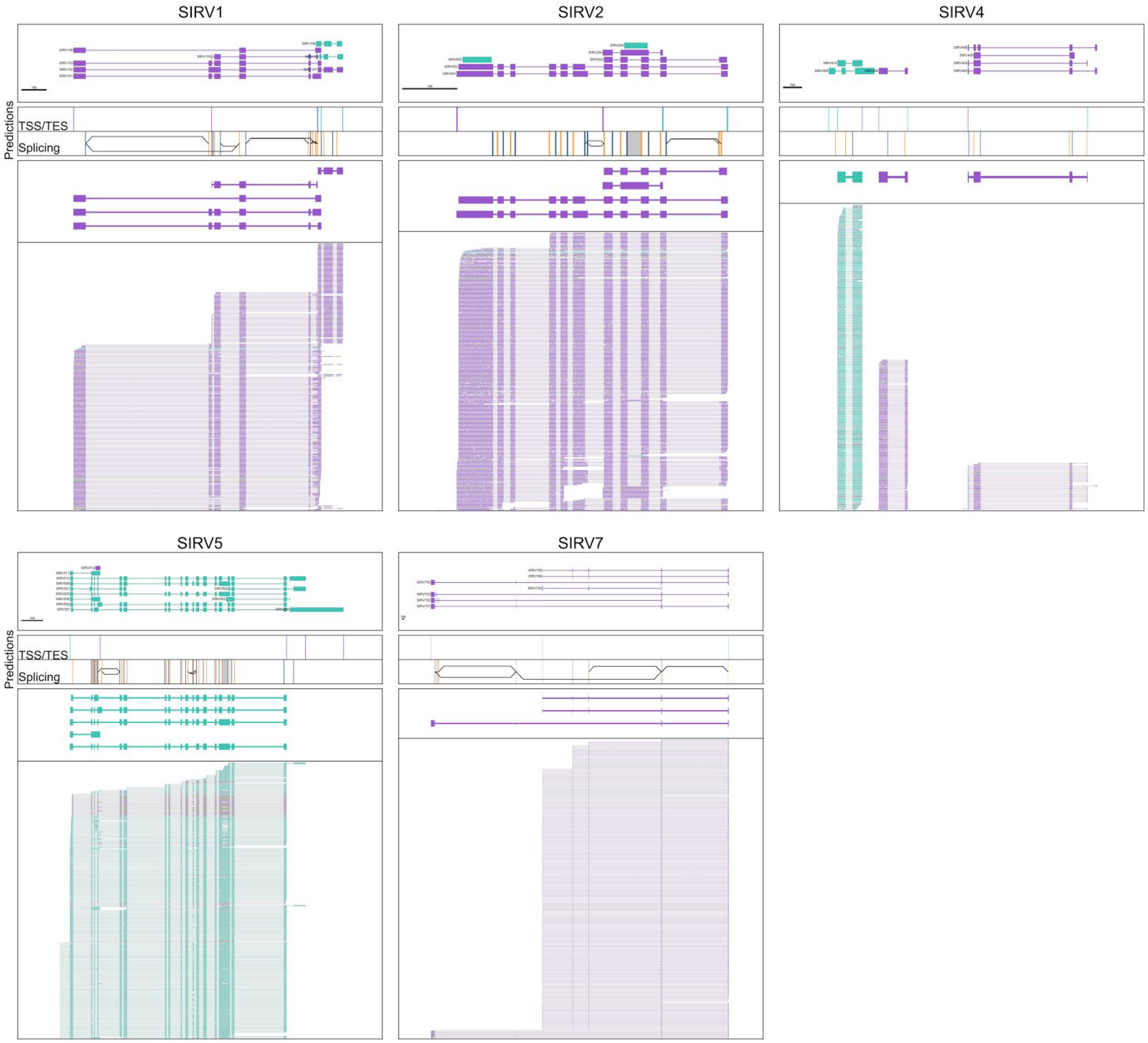

Supplementary Fig. 4: Identifying Transcript isoforms using ONT RNAseq
Genome Browser view of indicated SIRV gene loci. Top box contains transcript annotations, second and third box contain TSS (Teal) /TES (Purple) and splice sites (5’SS: yellow, 3’SS: blue) locations predicted from the read data, respectively. Black lines and grey areas in box 3 indicate alternative splicing and intron retention events predicted from the read data. Box 4 contains read alignments of isoform consensus reads. Box 5 contains ONT 2D read alignments. Direction of transcripts, isoform consensus, and ONT 2D reads are indicated by their color (Teal: 5’ to 3’, Purple: 3’ to 5’).

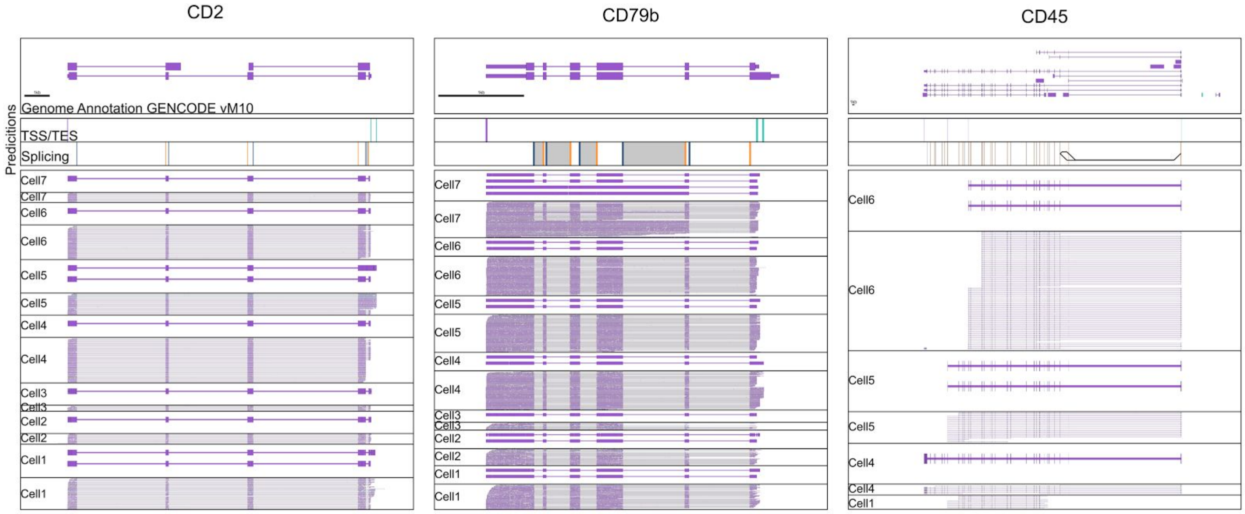

<u>Supplementary Fig. 5: Diverse Isoforms of B cell surface receptors identified using ONT RNAseq</u>

Genome Browser view of the indicated B cell surface receptor gene loci. Top box contains transcript annotations, second and third box contain TSS (Teal) /TES (Purple) and splice site (5'SS: yellow, 3'SS: blue) locations predicted, respectively. Black lines and gray areas in box 3 indicate alternative splicing and intron retention events predicted. Below boxes alternatingly contain read alignments of isoform consensus reads and ONT 2D read alignments. Direction of transcripts, isoform consensus, and ONT 2D reads are indicated by their color (Teal: 5' to 3', Purple: 3' to 5').

TSO_Smartseq2	AAGCAGTGGTATCAACGCAGAGTACATrGrGrG
Oligo-dT30VN-smartseq2-iso	/5Me-isodC/AAGCAGTGGTATCAACGCAGAGTACTTTTTTTTTTTTTTTTTTTTTTTTTTTTTTVN
ISPCR_Primer	AAGCAGTGGTATCAACGCAGAGT
Tn5ME-R	[phos]CTGTCTCTTATACACATCT
Tn5ME-A	TCGTCGGCAGCGTCAGATGTGTATAAGAGACAG
Tn5ME-B	GTCTCGTGGGCTCGGAGATGTGTATAAGAGACAG
Nextera_Primer_A	AATGATACGGCGACCACCGAGATCTACAC [i5] TCGTCGGCAGCGTCAGATG
Nextera_Primer_B	CAAGCAGAAGACGGCATACGAGAT [i7] GTCTCGTGGGCTCGGAGATGTGTAT
Nextera_Primer_A_Universal	AATGATACGGCGACCACCGAGATCTACAC
ONT_Index1_ISPCR	TGATCGT GATGCAGTGCTGCTCTTATCATATTTGTATTGACGACAGCCGCCTTCGCGGTTTCCTCAG AAGCAGTGGTATCAACGCAGAGT
ONT_Index2_ISPCR	TGATCGT ACATTTAAAAATAAAGGCTTATTGTAGGCAGAGGTACGCCCTTTTAGTGGCTGCGGTAAA AAGCAGTGGTATCAACGCAGAGT
ONT_Index3_ISPCR	TGATCGT ATATCTTCGGATCCCTTTGTCTAACCAAATTAATCGAATTCTCTCATTTAAGACCTTAAT AAGCAGTGGTATCAACGCAGAGT
ONT_Index4_ISPCR	TGATCGT TTTTGTATCGCAAGGTACTCCCGATCTTAATGGATGGCCGGAAGTGGTACGGATGCAATA AAGCAGTGGTATCAACGCAGAGT
ONT_Index5_ISPCR	TGATCGT AGCGCGGGTGAGAGGGTAATTAGGCGCGTTCACCTACGCTACGCTAACGGGCGATTCTAT AAGCAGTGGTATCAACGCAGAGT
ONT_Index6_ISPCR	TGATCGT CCTACTATCCCTAAGCGCATATCTCGCGCAGTAGCCTCCGAATATGTCGGCATCTGATGT AAGCAGTGGTATCAACGCAGAGT
ONT_Index7_ISPCR	TGATCGT TACCCAGGTTGAGTTAGTGTTGAGCTCACGGAACTTATTGTATGAGTAGAGATTTGTAAG AAGCAGTGGTATCAACGCAGAGT
ONT_Index8_ISPCR	TGATCGT AGCTGTTAGTTAGCTCGCTCAGCTAATAGTTGCCCACACAACGTCAAAATTAGAGAACGG AAGCAGTGGTATCAACGCAGAGT
ONT_Index9_ISPCR	TGATCGT TCGGAGGTTTTACCATACCACAGAATTAACCGCATCCATCAGGACCAAACGAGCGAAGCC AAGCAGTGGTATCAACGCAGAGT
ONT_Index10_ISPCR	TGATCGT GACCTACTTGGCAAACTTAGTAGGAAATATAGCTCTAAGGCATATATAGAAATACCATCG AAGCAGTGGTATCAACGCAGAGT

Supplementary Table 1: List of Oligos

All oligos used in the study are shown.

1.) Spaces in sequences are for visual emphasis and do not indicate gaps in the sequences

2.) Nextera_Primer_A and Nextera_Primer_B represent groups of sequences

3.) [i5] and [i7] indicate different Illumina index sequences

		Illumina Reads		ONT 2D Reads				Percent of pass alignment filter ONT 2D reads		
Technology	Sample	Raw Reads	Aligned	Pass Filter	Fail Filter	Analyzed	Pass Alignment Filter	Complete Reads: Both ISPCR sequences found	1st ISPCR sequence found	2nd ISPCR sequence found
R7.3	Cell1	351876	321251	23957	28739	52696	32556	38.16	71.71	55.91
R7.3	Cell2	185258	157782	8665	13396	22061	13889	36.85	69.00	57.38
R7.3	Cell3	109712	89998	5081	12668	17749	8500	36.27	67.86	56.29
R9.4	Cell4	121586	94237	76721	1936	76721	74604	62.32	76.51	81.29
R9.4	Cell5	174128	158788	53651	4223	53651	52304	66.47	79.11	84.10
R9.4	Cell6	97314	89572	127408	1318	127408	124007	66.50	79.99	83.20
R9.4	Cell7	73086	59738	105056	2432	105056	102412	64.01	76.31	83.89
R9.4	Lexogen_10fg_Rep1	--	--	12725	368	12725	10837	68.11	80.37	84.47
R9.4	Lexogen_10fg_Rep2	--	--	17915	412	17915	15327	70.94	82.10	86.12
R9.4	Lexogen_100fg_Rep1	--	--	5367	526	5367	4770	66.98	79.87	83.63
R9.4	Lexogen_100fg_Rep2	--	--	5745	528	5745	5197	65.25	78.01	83.41

Supplementary Table 2: Illumina and ONT Read Numbers.

Illumina Reads: Numbers indicate indivdual reads, not read pairs. "Aligned" Reads are reads succesfully aligned using STAR.

ONT 2D Reads: "Pass Filter" and "Fail Filter" Reads are determined by the Metrichor software based on quality scores.

"Pass Alignment Filter" Reads were aligned using BLAT and more than 60% of their bases aligned to the genome.

Complete reads as mentioned in the manuscript are defined as reads for which both ISPCR sequences could be identified and trimmed.

	Median read count		p-value (MWU one-sided)
Alternative 5' SS	81	42.5	0.0003
Alternative 3' SS	78	33	0.0019
Intron Retention	15	10.5	0.0412
	yes	no	
	Illumina supported		

Supplementary Table 3: Alternative Splice Sites.
Median ONT 2D read count is shown for alternative splice sites. Splice sites are separated into sites with and without Illumina read support. p-values are calculated using the scipy.stats.mannwhitneyu function with keyword argument alternative='greater'
