# Supplementary material for: Nanopore long-read RNAseq reveals widespread transcriptional variation among the surface receptors of individual B cells
Source: Nat Commun. 2017 Jul 19;8:16027. doi: 10.1038/ncomms16027 (PMC5524981; doi:10.1038/ncomms16027)
Supplement: Peer Review File [file ncomms16027-s2.pdf]

Editorial Note: This manuscript has been previously reviewed at another journal that is not operating a transparent peer review scheme. This document only contains reviewer comments and rebuttal letters for versions considered at Nature Communications.

REVIEWERS' COMMENTS:

Reviewer #1 (Remarks to the Author):

I'm satisfied with the authors responses to my comments.
